# 2-(4-Bromo­phen­yl)-5-fluoro-3-methyl­sulfinyl-1-benzofuran

**DOI:** 10.1107/S1600536809030190

**Published:** 2009-08-08

**Authors:** Hong Dae Choi, Pil Ja Seo, Byeng Wha Son, Uk Lee

**Affiliations:** a Department of Chemistry, Dongeui University, San 24 Kaya-dong Busanjin-gu, Busan 614-714, Republic of Korea; b Department of Chemistry, Pukyong National University, 599-1 Daeyeon 3-dong, Nam-gu, Busan 608-737, Republic of Korea

## Abstract

In the title compound, C_15_H_10_BrFO_2_S, the O atom and the methyl group of the methyl­sulfinyl substituent lie on opposite sides of the plane through the benzofuran fragment. The 4-bromo­phenyl ring is rotated out of the benzofuran plane [dihedral angle = 38.98 (8)°], while the structure is stabilized by an inter­molecular C—H⋯O hydrogen bond and a Br⋯O halogen bond [3.036 (2) Å] and has an inter­molecular C–H⋯π inter­action between the 4-bromo­phenyl H atom and the benzene ring of an adjacent benzofuran mol­ecule, as well as aromatic π–π inter­actions between the benzene rings of the benzofuran systems [centroid–centroid distance = 3.482 (3) Å].

## Related literature

For the crystal structures of similar 2-(4-bromo­phen­yl)-3-methyl­sulfinyl-1-benzofuran derivatives, see: Choi *et al.* (2007*a* ▶,*b* ▶). For the pharmacological activity of benzofuran compounds, see: Howlett *et al.* (1999 ▶); Twyman & Allsop (1999 ▶). For a review of halogen bonding, see: Politzer *et al.* (2007 ▶).
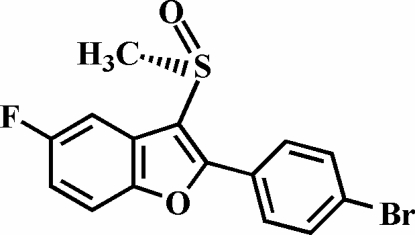


## Experimental

### 

#### Crystal data

C_15_H_10_BrFO_2_S
*M* *_r_* = 353.20
Triclinic, 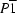

*a* = 8.6909 (7) Å
*b* = 9.1765 (7) Å
*c* = 10.1308 (8) Å
α = 105.989 (1)°
β = 114.811 (1)°
γ = 99.423 (1)°
*V* = 667.91 (9) Å^3^
*Z* = 2
Mo *K*α radiation
μ = 3.24 mm^−1^
*T* = 293 K
0.40 × 0.20 × 0.10 mm

#### Data collection

Bruker SMART CCD-detector diffractometer
Absorption correction: multi-scan (*SADABS*; Sheldrick, 1996 ▶) *T*_min_ = 0.461, *T*_max_ = 0.720
5792 measured reflections
2861 independent reflections
2589 reflections with *I* > 2σ(*I*)
*R*_int_ = 0.016

#### Refinement

*R*[*F* ^2^ > 2σ(*F* ^2^)] = 0.024
*wR*(*F* ^2^) = 0.062
*S* = 1.05
2861 reflections
182 parameters
H-atom parameters constrained
Δρ_max_ = 0.56 e Å^−3^
Δρ_min_ = −0.52 e Å^−3^

### 

Data collection: *SMART* (Bruker, 2001 ▶); cell refinement: *SAINT* (Bruker, 2001 ▶); data reduction: *SAINT*; program(s) used to solve structure: *SHELXS97* (Sheldrick, 2008 ▶); program(s) used to refine structure: *SHELXL97* (Sheldrick, 2008 ▶); molecular graphics: *ORTEP-3* (Farrugia, 1997 ▶) and *DIAMOND* (Brandenburg, 1998 ▶); software used to prepare material for publication: *SHELXL97*.

## Supplementary Material

Crystal structure: contains datablocks global, I. DOI: 10.1107/S1600536809030190/zs2004sup1.cif

Structure factors: contains datablocks I. DOI: 10.1107/S1600536809030190/zs2004Isup2.hkl

Additional supplementary materials:  crystallographic information; 3D view; checkCIF report

## Figures and Tables

**Table 1:** Hydrogen-bond geometry (Å, °)

*D*—H⋯*A*	*D*—H	H⋯*A*	*D*⋯*A*	*D*—H⋯*A*
C15—H15*C*⋯O2^i^	0.96	2.36	3.251 (3)	155
C13—H13⋯*Cg*^ii^	0.93	2.74	3.366 (3)	125

Symmetry codes: (i) 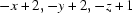; (ii) 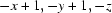.

**Fig. 1.**
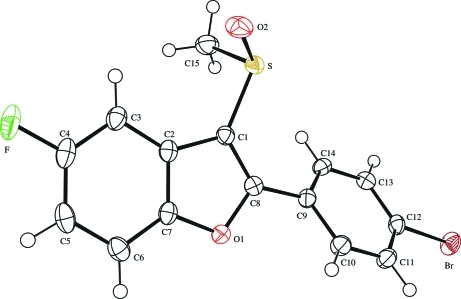
The molecular structure of the title compound with the atom numbering scheme. Displacement ellipsoids are drawn at the 50% probability level.

**Fig. 2.**
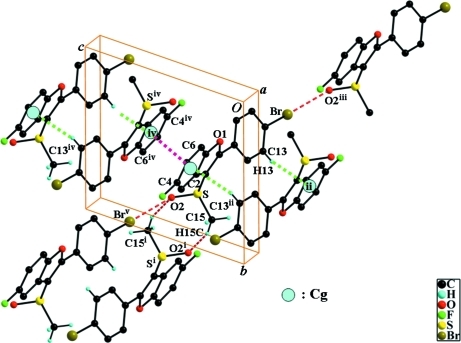
C–H···O, C–Br···O, C–H···π, and π–π interactions (dotted lines) in the crystal structure of title compound. Cg denotes the ring centroid. [Symmetry codes: (i) - x + 2, - y + 2, - z + 1; (ii) - x + 1, - y + 1, - z; (iii) x, y - 1, z - 1; (iv) - x + 1, - y + 1, - z + 1 (v) x, y + 1, z + 1]. For other codes, see Table 1.

## supplementary crystallographic information

### Comment

Benzofuran derivatives are of considerable interest because of their pharmacological properties (Howlett et al., 1999; Twyman & Allsop, 1999). This present work is related to our communications on the synthesis and structures of 2-(4-bromophenyl)-3-methylsulfinyl-1-benzofuran analogues, viz. 2-(4-bromophenyl)-5-methyl-3-methylsulfinyl-1-benzofuran (Choi et al., 2007a) and 2-(4-bromophenyl)-5,7-dimethyl-3-methylsulfinyl-1-benzofuran (Choi et al., 2007b). Here we report the crystal structure of the title compound (I) (Fig. 1). The benzofuran unit in (I) is essentially planar, with a mean deviation of 0.006 (2) Å from the least-squares plane defined by the nine constituent atoms. The dihedral angle formed by the planes of the benzofuran and the 4-bromophenyl rings is 38.98 (8) °. The crystal packing (Fig. 2) is stabilized by an intermolecular C–H···O hydrogen bond between the methyl H atom and the S═O unit (Table 1) and a Br···O halogen bond [Br···O2^iii^, 3.036 (2) Å; C–Br···O, 165.46 (7) °] (symmetry code : (iii) x, y-1, z-1) (Politzer et al.,  2007). The crystal packing also has an intermolecular C–H···π interaction between a 4-bromophenyl H atom (C13) and the benzene ring of an adjacent molecule [C–H···Cg^ii^] (Table 1), (where Cg is the centroid of the C2-C7 benzene ring). Further stability comes from aromatic π–π interactions between the benzene rings of the adjacent molecules, with a Cg···Cg^iv^ distance of 3.482 (3) Å [symmetry code: (iv) -x + 1, -y + 1, -z + 1].

### Experimental

3-Chloroperoxybenzoic acid (77%) (291 mg, 1.3 mmol) was added in small portions to a stirred solution of 2-(4-bromophenyl)-5-fluoro-3-methylsulfanyl-1-benzofuran (310 mg, 1.2 mmol) in dichloromethane (30 mL) at 273 K. After being stirred at room temperature for 3h, the mixture was washed with saturated sodium bicarbonate solution and the organic layer was separated, dried over magnesium sulfate, filtered and concentrated in vacuum. The residue was purified by column chromatography [hexane-ethyl acetate, 1 : 2 (v/v)] to afford the title compound as a colorless solid [yield 81%, m.p. 442-443 K; *R*_f_ = 0.68 (hexane-ethyl acetate, 1 : 2 (v/v)]. Single crystals suitable for X-ray diffraction were prepared by slow evaporation of a solution of the title compound in tetrahydrofuran at room temperature.

### Refinement

All H atoms were positioned geometrically and refined using a riding model, with C–H(aromatic) = 0.93 Å C–H (aliphatic) = 0.96 Å. and with *U*_iso_(H) = 1.2*U*_eq_(C) (aromatic) H atoms and 1.5 *U*_eq_(C) (aliphatic).

### Crystal data

**Table:**

C_15_H_10_BrFO_2_S	*Z* = 2
*M**_r_* = 353.20	*F*(000) = 352
Triclinic, *P*1	*D*_x_ = 1.756 Mg m^−^^3^
Hall symbol: -P 1	Melting point = 442–443 K
*a* = 8.6909 (7) Å	Mo *K*α radiation, λ = 0.71073 Å
*b* = 9.1765 (7) Å	Cell parameters from 4045 reflections
*c* = 10.1308 (8) Å	θ = 2.4–27.5°
α = 105.989 (1)°	µ = 3.24 mm^−^^1^
β = 114.811 (1)°	*T* = 293 K
γ = 99.423 (1)°	Block, colorless
*V* = 667.91 (9) Å^3^	0.40 × 0.20 × 0.10 mm

### Data collection

**Table:**

Bruker SMART CCD-detector diffractometer	2861 independent reflections
Radiation source: fine-focus sealed tube	2589 reflections with *I* > 2σ(*I*)
graphite	*R*_int_ = 0.016
Detector resolution: 10.0 pixels mm^-1^	θ_max_ = 27.0°, θ_min_ = 2.4°
φ and ω scans	*h* = −11→11
Absorption correction: multi-scan (*SADABS*; Sheldrick, 1996)	*k* = −11→11
*T*_min_ = 0.461, *T*_max_ = 0.720	*l* = −12→12
5792 measured reflections	

### Refinement

**Table:**

Refinement on *F*^2^	Primary atom site location: structure-invariant direct methods
Least-squares matrix: full	Secondary atom site location: difference Fourier map
*R*[*F*^2^ > 2σ(*F*^2^)] = 0.024	Hydrogen site location: difference Fourier map
*wR*(*F*^2^) = 0.062	H-atom parameters constrained
*S* = 1.05	*w* = 1/[σ^2^(*F*_o_^2^) + (0.0263*P*)^2^ + 0.5235*P*] where *P* = (*F*_o_^2^ + 2*F*_c_^2^)/3
2861 reflections	(Δ/σ)_max_ = 0.001
182 parameters	Δρ_max_ = 0.56 e Å^−^^3^
0 restraints	Δρ_min_ = −0.52 e Å^−^^3^

### Special details

**Table:**

Geometry. All esds (except the esd in the dihedral angle between two l.s. planes) are estimated using the full covariance matrix. The cell esds are taken into account individually in the estimation of esds in distances, angles and torsion angles; correlations between esds in cell parameters are only used when they are defined by crystal symmetry. An approximate (isotropic) treatment of cell esds is used for estimating esds involving l.s. planes.
Refinement. Refinement of F^2^ against ALL reflections. The weighted R-factor wR and goodness of fit S are based on F^2^, conventional R-factors R are based on F, with F set to zero for negative F^2^. The threshold expression of F^2^ > 2sigma(F^2^) is used only for calculating R-factors(gt) etc. and is not relevant to the choice of reflections for refinement. R-factors based on F^2^ are statistically about twice as large as those based on F, and R- factors based on ALL data will be even larger.

### Fractional atomic coordinates and isotropic or equivalent isotropic displacement parameters (Å^2^)

**Table:**

	*x*	*y*	*z*	*U*_iso_*/*U*_eq_	
Br	0.87777 (3)	0.07461 (3)	−0.19442 (3)	0.03165 (8)	
S	0.83195 (6)	0.75073 (6)	0.37122 (6)	0.02170 (11)	
F	0.27524 (19)	0.78610 (17)	0.54077 (16)	0.0402 (3)	
O1	0.44103 (18)	0.34213 (15)	0.19174 (16)	0.0212 (3)	
O2	0.8984 (2)	0.83704 (18)	0.54254 (17)	0.0315 (3)	
C1	0.6353 (2)	0.5929 (2)	0.3021 (2)	0.0194 (4)	
C2	0.4984 (3)	0.5994 (2)	0.3476 (2)	0.0200 (4)	
C3	0.4642 (3)	0.7204 (3)	0.4402 (2)	0.0249 (4)	
H3	0.5391	0.8260	0.4910	0.030*	
C4	0.3134 (3)	0.6731 (3)	0.4513 (2)	0.0278 (4)	
C5	0.1973 (3)	0.5170 (3)	0.3795 (3)	0.0285 (5)	
H5	0.0969	0.4935	0.3914	0.034*	
C6	0.2324 (3)	0.3962 (3)	0.2896 (2)	0.0262 (4)	
H6	0.1586	0.2903	0.2410	0.031*	
C7	0.3836 (3)	0.4430 (2)	0.2768 (2)	0.0210 (4)	
C8	0.5949 (3)	0.4379 (2)	0.2100 (2)	0.0196 (4)	
C9	0.6748 (2)	0.3554 (2)	0.1231 (2)	0.0194 (4)	
C10	0.6747 (3)	0.1994 (2)	0.1089 (2)	0.0236 (4)	
H10	0.6313	0.1507	0.1612	0.028*	
C11	0.7387 (3)	0.1167 (2)	0.0176 (2)	0.0254 (4)	
H11	0.7386	0.0130	0.0083	0.031*	
C12	0.8029 (3)	0.1910 (2)	−0.0596 (2)	0.0219 (4)	
C13	0.8077 (3)	0.3461 (2)	−0.0451 (2)	0.0217 (4)	
H13	0.8534	0.3948	−0.0961	0.026*	
C14	0.7437 (3)	0.4286 (2)	0.0465 (2)	0.0212 (4)	
H14	0.7466	0.5330	0.0570	0.025*	
C15	0.7267 (3)	0.8706 (3)	0.2720 (3)	0.0310 (5)	
H15A	0.6331	0.8880	0.2950	0.047*	
H15B	0.6768	0.8163	0.1604	0.047*	
H15C	0.8138	0.9718	0.3075	0.047*	

### Atomic displacement parameters (Å^2^)

**Table:**

	*U*^11^	*U*^22^	*U*^33^	*U*^12^	*U*^13^	*U*^23^
Br	0.04037 (14)	0.03169 (13)	0.03461 (13)	0.01806 (10)	0.02714 (11)	0.01103 (10)
S	0.0186 (2)	0.0212 (2)	0.0202 (2)	0.00384 (18)	0.00880 (19)	0.00360 (19)
F	0.0428 (8)	0.0511 (9)	0.0360 (7)	0.0286 (7)	0.0263 (7)	0.0104 (6)
O1	0.0217 (7)	0.0186 (6)	0.0234 (7)	0.0064 (5)	0.0122 (6)	0.0062 (5)
O2	0.0288 (8)	0.0312 (8)	0.0212 (7)	−0.0001 (6)	0.0108 (6)	−0.0005 (6)
C1	0.0179 (9)	0.0206 (9)	0.0180 (9)	0.0065 (7)	0.0077 (7)	0.0065 (7)
C2	0.0199 (9)	0.0245 (10)	0.0172 (9)	0.0102 (8)	0.0086 (7)	0.0093 (8)
C3	0.0252 (10)	0.0266 (10)	0.0199 (10)	0.0111 (8)	0.0098 (8)	0.0053 (8)
C4	0.0306 (11)	0.0392 (12)	0.0200 (10)	0.0220 (10)	0.0137 (9)	0.0119 (9)
C5	0.0233 (10)	0.0449 (13)	0.0281 (11)	0.0186 (9)	0.0150 (9)	0.0206 (10)
C6	0.0221 (10)	0.0327 (11)	0.0276 (10)	0.0097 (8)	0.0123 (9)	0.0160 (9)
C7	0.0220 (10)	0.0253 (10)	0.0183 (9)	0.0119 (8)	0.0096 (8)	0.0098 (8)
C8	0.0192 (9)	0.0215 (9)	0.0185 (9)	0.0068 (7)	0.0088 (7)	0.0087 (7)
C9	0.0186 (9)	0.0206 (9)	0.0165 (9)	0.0070 (7)	0.0075 (7)	0.0053 (7)
C10	0.0266 (10)	0.0231 (10)	0.0253 (10)	0.0082 (8)	0.0153 (9)	0.0106 (8)
C11	0.0298 (11)	0.0204 (9)	0.0299 (11)	0.0106 (8)	0.0170 (9)	0.0095 (8)
C12	0.0208 (9)	0.0246 (10)	0.0199 (9)	0.0096 (8)	0.0112 (8)	0.0050 (8)
C13	0.0210 (9)	0.0238 (10)	0.0183 (9)	0.0046 (8)	0.0095 (8)	0.0072 (8)
C14	0.0229 (10)	0.0175 (9)	0.0196 (9)	0.0060 (7)	0.0084 (8)	0.0057 (7)
C15	0.0287 (11)	0.0232 (10)	0.0358 (12)	0.0059 (9)	0.0115 (10)	0.0121 (9)

### Geometric parameters (Å, °)

**Table:**

Br—C12	1.897 (2)	C6—C7	1.384 (3)
Br—O2^i^	3.036 (2)	C6—H6	0.9300
S—C1	1.771 (2)	C8—C9	1.466 (3)
S—C15	1.796 (2)	C9—C14	1.398 (3)
F—C4	1.361 (2)	C9—C10	1.398 (3)
O1—C8	1.383 (2)	C10—C11	1.388 (3)
O1—C7	1.386 (2)	C10—H10	0.9300
C1—C8	1.359 (3)	C11—C12	1.387 (3)
C1—C2	1.449 (3)	C11—H11	0.9300
C2—C7	1.391 (3)	C12—C13	1.382 (3)
C2—C3	1.404 (3)	C13—C14	1.388 (3)
C3—C4	1.373 (3)	C13—H13	0.9300
C3—H3	0.9300	C14—H14	0.9300
C4—C5	1.391 (3)	C15—H15A	0.9600
C5—C6	1.392 (3)	C15—H15B	0.9600
C5—H5	0.9300	C15—H15C	0.9600
			
C12—Br—O2^i^	165.46 (7)	C1—C8—C9	133.66 (18)
O2—S—C1	106.07 (9)	O1—C8—C9	115.03 (16)
O2—S—C15	107.01 (10)	C14—C9—C10	119.19 (18)
C1—S—C15	97.48 (10)	C14—C9—C8	120.39 (17)
C8—O1—C7	105.92 (14)	C10—C9—C8	120.34 (17)
C8—C1—C2	106.94 (17)	C11—C10—C9	120.58 (18)
C8—C1—S	125.96 (15)	C11—C10—H10	119.7
C2—C1—S	126.73 (15)	C9—C10—H10	119.7
C7—C2—C3	119.59 (18)	C12—C11—C10	119.00 (18)
C7—C2—C1	105.25 (16)	C12—C11—H11	120.5
C3—C2—C1	135.15 (19)	C10—C11—H11	120.5
C4—C3—C2	115.79 (19)	C13—C12—C11	121.53 (18)
C4—C3—H3	122.1	C13—C12—Br	119.15 (15)
C2—C3—H3	122.1	C11—C12—Br	119.28 (15)
F—C4—C3	117.9 (2)	C12—C13—C14	119.28 (18)
F—C4—C5	117.34 (19)	C12—C13—H13	120.4
C3—C4—C5	124.73 (19)	C14—C13—H13	120.4
C4—C5—C6	119.66 (19)	C13—C14—C9	120.40 (18)
C4—C5—H5	120.2	C13—C14—H14	119.8
C6—C5—H5	120.2	C9—C14—H14	119.8
C7—C6—C5	116.0 (2)	S—C15—H15A	109.5
C7—C6—H6	122.0	S—C15—H15B	109.5
C5—C6—H6	122.0	H15A—C15—H15B	109.5
C6—C7—O1	125.17 (18)	S—C15—H15C	109.5
C6—C7—C2	124.19 (18)	H15A—C15—H15C	109.5
O1—C7—C2	110.64 (16)	H15B—C15—H15C	109.5
C1—C8—O1	111.24 (16)		
			
O2—S—C1—C8	−132.93 (17)	C1—C2—C7—O1	0.0 (2)
C15—S—C1—C8	116.90 (18)	C2—C1—C8—O1	0.0 (2)
O2—S—C1—C2	39.08 (19)	S—C1—C8—O1	173.31 (13)
C15—S—C1—C2	−71.09 (18)	C2—C1—C8—C9	176.90 (19)
C8—C1—C2—C7	0.0 (2)	S—C1—C8—C9	−9.8 (3)
S—C1—C2—C7	−173.23 (15)	C7—O1—C8—C1	0.0 (2)
C8—C1—C2—C3	179.5 (2)	C7—O1—C8—C9	−177.54 (15)
S—C1—C2—C3	6.3 (3)	C1—C8—C9—C14	−37.8 (3)
C7—C2—C3—C4	−1.2 (3)	O1—C8—C9—C14	139.07 (18)
C1—C2—C3—C4	179.4 (2)	C1—C8—C9—C10	145.6 (2)
C2—C3—C4—F	179.60 (17)	O1—C8—C9—C10	−37.6 (2)
C2—C3—C4—C5	0.6 (3)	C14—C9—C10—C11	−1.3 (3)
F—C4—C5—C6	−178.56 (18)	C8—C9—C10—C11	175.46 (18)
C3—C4—C5—C6	0.4 (3)	C9—C10—C11—C12	0.0 (3)
C4—C5—C6—C7	−0.8 (3)	C10—C11—C12—C13	1.3 (3)
C5—C6—C7—O1	−179.26 (17)	C10—C11—C12—Br	−176.36 (15)
C5—C6—C7—C2	0.2 (3)	C11—C12—C13—C14	−1.3 (3)
C8—O1—C7—C6	179.56 (19)	Br—C12—C13—C14	176.41 (14)
C8—O1—C7—C2	0.0 (2)	C12—C13—C14—C9	−0.1 (3)
C3—C2—C7—C6	0.8 (3)	C10—C9—C14—C13	1.3 (3)
C1—C2—C7—C6	−179.57 (18)	C8—C9—C14—C13	−175.41 (17)
C3—C2—C7—O1	−179.62 (16)		

Symmetry codes: (i) *x*, *y*−1, *z*−1.

### Hydrogen-bond geometry (Å, °)

**Table:**

*D*—H···*A*	*D*—H	H···*A*	*D*···*A*	*D*—H···*A*
C15—H15C···O2^ii^	0.96	2.36	3.251 (3)	155
C13—H13···Cg^iii^	0.93	2.74	3.366 (3)	125

Symmetry codes: (ii) −*x*+2, −*y*+2, −*z*+1; (iii) −*x*+1, −*y*+1, −*z*.
